# Qigong Versus Usual Exercise in the Treatment of Chronic Nonspecific Low Back Pain as an Add-On to a Standardized Physiotherapy Program

**DOI:** 10.7759/cureus.81492

**Published:** 2025-03-31

**Authors:** Spyridon Sotiropoulos, Theodora Plavoukou, George Georgoudis

**Affiliations:** 1 Physiotherapy Department and Musculoskeletal Physiotherapy Research Laboratory, University of West Attica (UNIWA), Athens, GRC

**Keywords:** chronic low back pain, kinesiophobia, physiotherapy, proprioception, qigong, rehabilitation, strengthening exercises

## Abstract

Introduction: Chronic nonspecific low back pain (CNSLBP) is a leading cause of disability worldwide. Exercise-based interventions, particularly strengthening exercises, are widely used in rehabilitation. However, mind-body approaches such as Qigong, which integrate movement, breath control, and mindfulness, may offer additional psychological benefits. Despite evidence supporting Qigong in pain management, its effectiveness as an adjunct to physiotherapy remains unclear. This study compared the effects of Qigong versus strengthening exercises, integrated into a standardized physiotherapy program, on pain perception, disability, kinesiophobia, and proprioception in CNSLBP patients.

Methods: A randomized controlled trial (RCT) was conducted with 42 participants who were assigned to either a Qigong combined with physiotherapy group, or a strengthening exercise combined with physiotherapy group for a duration of four weeks. Pain (Short-Form McGill Pain Questionnaire, SFMPQ), disability (Greek Roland Morris Disability Questionnaire, RMDQ), kinesiophobia (Greek Tampa Scale of Kinesiophobia, TSK), and proprioception (sway-length on a baropodometer) were assessed pre- and post-intervention. Appropriate statistical analyses were conducted for within- and between-group comparisons. Statistical significance was set at p < 0.05.

Results: Both groups showed significant within-group improvements in pain perception, disability, kinesiophobia, and proprioception (p<0.05). However, no statistically significant between-group differences were observed. A trend toward greater kinesiophobia reduction in the Qigong group (p=0.069) suggests a potential psychological benefit.

Discussion: Qigong and strengthening exercises, when combined with physiotherapy, are equally effective in improving CNSLBP symptoms. Future research should explore longer interventions (>12 weeks) and larger trials to determine whether Qigong offers distinct advantages over conventional exercise programs.

## Introduction

Low back pain (LBP) is a pervasive global health issue, significantly contributing to disability and impacting the quality of life of those affected [1]. The prevalence of LBP has increased fivefold over 15 years, primarily due to rising obesity and sedentary lifestyles [2]. Chronic nonspecific low back pain (CNSLBP) accounts for 90-95% of cases, with a prevalence of 18%. The term chronic is used when the duration of the symptoms is more than three months [3].

Usually, a number of approaches, including pharmacological approaches, physiotherapy and exercise, have been used [4]. Many different types of exercises have been reported to be beneficial in pain and disability [5]. In particular strengthening and stretching exercises have proven to be effective in reducing pain and improving quality of life [6]. Among these exercise-based approaches, Qigong exercises, offer a holistic practice that combines gentle movements including both stretching and strengthening elements, breath control, and mindfulness, which may provide additional benefits for managing pain and improving overall well-being.

Qigong is a mind and body type of exercise that has been used as a treatment option for a variety of diseases in the context of traditional Chinese medicine including CNSLBP [7]. Recent studies have shown that Qigong - perhaps due to its holistic approach encompassing biological and psychological elements as a treatment technique - has been effective as a treatment for patients suffering with low back pain [8]. However, its benefit as an adjunct to a physiotherapy program has not been investigated.

This study investigates the combined effect of Qigong exercises and standardized physiotherapy compared to commonly used exercises integrated into the same physiotherapy program. To our knowledge, no research has yet addressed this specific topic. Therefore, the findings could offer valuable initial insights into the potential advantages of integrating Qigong exercises into clinical practice. We hypothesized that Qigong combined with physiotherapy would lead to greater improvements in pain, disability, kinesiophobia, and proprioception than strengthening exercises combined with physiotherapy. The objective of this study was to compare the effects of these two interventions on these outcomes in individuals with CNSLBP.

## Materials and methods

Design

This trial was designed as a two-arm, single-blind randomised controlled trial (RCT) conducted at the Aretaieion Hospital Pain Clinic and was registered in the ISRCTN registry (ISRCTN15963005). The study consisted of two experimental groups suffering from CNSLBP. Both groups followed a standardized usual physiotherapy program with group A receiving a strengthening exercise program, while group B received a standardized Qigong program. The total duration of treatment was four weeks, with two treatment sessions per week (eight treatment sessions in total).

Sample size calculations

Sample size calculation was determined by the G-power analysis test based on the primary outcome measure regarding pain, the Short-Form McGill Pain Questionnaire (SFMPQ). Based on the literature, the sample calculation was based on the effect size of the primary tool of SFMPQ in patients with lumbar pain (effect size=0.8), with an α=0.05 and a sample of a total number of 42 subjects was determined, providing a power of 0.816. 

Participants

**Figure 1:**
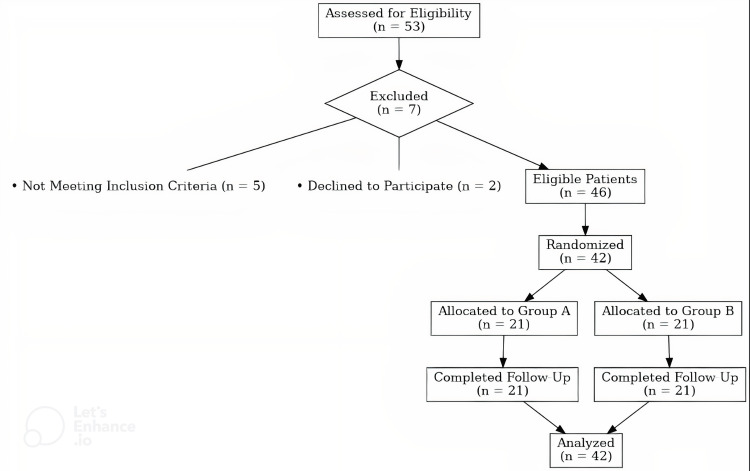
Intention to treat flowchart

Initially, 53 patients were assessed for eligibility from the waiting list of the Aretaieion Hospital Pain clinic. Of these, seven participants were excluded, with five not meeting the inclusion criteria and two declining to participate, resulting in 46 eligible patients. From this pool, 42 participants were randomized into two equal groups of N=21 in each group (Table 1). The selection procedure can be found in Figure 1.

**Table 1:** Sample characteristics BMI: Body Mass Index, χ²: Chi-Square, U: Mann-Whitney U

-	Total Ν=42 Ν(%)	Group Α (Qigong) Ν=21 N(%)	Group B (Strengthening) Ν=21 N(%)	Statistical test	Significance level
Gender					
Male	17(40.5%)	6(28.6%)	11(52.4%)	χ² = 1.58	NS (p=0.116)
Female	25(59.5%)	15(71.4%)	10(47.6%)	-	
ΒΜΙ				χ² = 5.14	NS (p=0.086)
Normal	23(54.8%)	15(71.4%)	8(38.1%)		
Overweight	11(26.2%)	3(14.3%)	8(38.1%)		
Obese	8(19.0%)	3(14.3%)	5(23.8%)		
Occupation				χ² = 4.27	NS (p=0.162)
Employee	25(59.5%)	14(66.7%)	11(52.4%)		
Self-employed	9(21.4%)	2(9.5%)	7(33.3%)		
Retired	2(4.8%)	2(9.5%)	0(0.0%)		
Unemployed	6(14.3%)	3(14.3%)	3(14.3%)		
Type of Occupation				χ² = 2.99	NS (p=0.197)
Desk job	13(31.7%)	9(45.0%)	4(19.0%)		
Light manual labor	17(41.5%)	7(35.0%)	10(47.6%)		
Heavy manual labor	11(26.8%)	4(20.0%)	7(33.3%)		
Comorbidities				χ² = 1.14	NS (p=0.999)
None	28(66.7%)	14(66,7%)	14(66.7%)		
Yes	14(33.3%)	7(33,3%)	7(33.3%)		
Hypertension	7	4	3		
Osteoarthritis	1	0	1		
Other	6	3	3		
Age (Years)	44.5(11.7)	45.1(12.2)	43.9(11.4)	U = 237.5	NS (p=0.624)

The inclusion criteria for participation in the study required that individuals experience pain localized between the rib and the gluteal fold region. Eligible participants were required to be between 18 and 65 years of age and to have experienced symptoms for at least three months prior to enrollment. Additionally, all participants needed to provide informed consent and demonstrate proficiency in the Greek language.

Exclusion criteria included participation in an exercise rehabilitation or physiotherapy program for back pain within the past three months. Individuals with referred pain to the lower extremities due to neurological damage or those presenting with neurological signs and symptoms indicative of myelopathy were also excluded. Further exclusion criteria encompassed medical contraindications to exercise, systemic diseases such as rheumatoid arthritis, pregnancy, and the presence of other red flags, including fractures, malignancies, cauda equina syndrome, unexplained and rapid loss of muscle strength, and cardiorespiratory conditions. Finally, individuals with cognitive impairments or an inability to communicate were not eligible for participation.

Randomisation

Using simple randomization with a 1:1 ratio, 42 patients were assigned to two groups through the random.org software, which generated 21 unique numbers for each group. Participants received sealed envelopes prior to allocation, each containing a unique number corresponding to one of the two groups.

Blinding

The researcher taking the measurements had no knowledge of the patient group allocation. All communication was standardized on a predetermined script that the researcher had to follow. The participants would only present to the researcher their unique identification number initially allocated, without any further details regarding their personal information or group allocation.

Ethics

The trial was approved by the Ethics Committee of the National Kapodistrian University of Athens Medical School - Aretaeion Hospital (266/12-11-2020 - registration date 12th of November 2020). The study conforms to the Consort Guidelines [9]. 

Participants voluntarily took part in this study after they signed an informed consent and were notified that they could withdraw from the trial at any moment.

Procedures

Τhe intervention was delivered within four weeks for both groups and measurements were performed at baseline and immediately after the trial period.

Interventions

The standardized usual physiotherapy program consisted of high-frequency transcutaneous electrical nerve stimulation (TENS) with frequency of 100 Hz and wavelength of 100 ms for 15 minutes and radiofrequency treatment (TECAR therapy) for 20 minutes using the capacitive electrode (70 mm) for five minutes and subjective feeling of 3/10 on the Visual Analogue Scale (VAS) thermal perception scale and 15 minutes using the resistive electrode and subjective feeling of 4/10 on the VAS thermal perception scale. Both the resistive and the capacitive electrodes were operating at 500 Hz frequency.

The standard exercise program focused on strengthening the abdominals, dorsal muscles (extensors and the gluteal muscles, the abdominals, dorsal back), and gluteal muscles. It was organized into multiple stages, with difficulty gradually increasing across several sessions (refer to Appendix 1). 

Patients performed the exercises twice a week under supervision. They were instructed to repeat the protocol as a home exercise program for another two more times per week, following the written instructions provided through a leaflet. The exercise routine had an approximate duration of 20 minutes for each session.

The standardized Baduajin Qigong exercise routine was performed under supervision twice a week and each of the eight exercises was performed six times. The exercises followed the standard Baduajin Qigong sequence and included the exercises: Two Hands Hold up the Heavens, Drawing the Bow to Shoot the Hawk, Separate Heaven and Earth, Wise Owl Gazes Backwards or Look Back, Sway the Head and Shake the Tail, Two Hands Hold the Feet to Strengthen the Kidneys and Waist, Clench the Fists and Glare Fiercely and Bouncing on the Toes. 

Patients were instructed to perform the exercises at home two more times per week following the written instructions provided through a leaflet. The exercise routine had an approximate duration of 20 minutes for each session.

Outcome measurements

The measurements took place immediately before the beginning of the first treatment session (t1) and after each participant had concluded the intervention, he/she was assigned to (t2). 

The primary outcome measure for this study was the assessment of pain according to the SFMPQ Greek version. The SFMPQ was developed by Melzack [10] and the Greek version has shown good reliability and validity in detecting pain changes after treatment.

The Greek version of the Tampa Scale of Kinesiophobia (TSK) [11] was used to assess the kinesiophobia with good internal validity and reliability in assessing chronic spinal pain patients. To measure participants’ physical disability, the Greek version of the Roland Morris Disability Questionnaire (RMDQ) was used. RMDQ has been used extensively in previous studies investigating the effects of physiotherapy and exercises in patients with chronic low back pain [12]. The Greek version of the RMDQ has shown excellent internal consistency reliability and validity for the evaluation of Greek-speaking patients with low back pain [13]. 

Since proprioception is affected in patients with chronic musculoskeletal conditions including low back pain [14,15], the use of the Sway-length test on balance platforms has been proposed as a measurement tool [16]. In this study the baropodometric platform Freemed® with the software freeStep® v.1.4.01 (SensorMedica, Rome, Italy) was used to measure sway, by recording anteroposterior and lateral sway. Participants were instructed to maintain a neutral position firstly with their eyes open for 60 seconds and then with their eyes shut for the same amount of time. 

Statistical analysis

Statistical analysis was performed with SPSS (version 25.0; IBM Corp., Armonk, NY, USA) and STATA (version 17; StataCorp., College Station, TX, USA) software. Qualitative variables were summarized as absolute and relative frequencies (%), while quantitative variables were reported as mean, standard deviation (SD), median, and interquartile range (IQR). The normality of quantitative data was evaluated graphically through histograms and assessed using the Shapiro-Wilk test. The Chi-square test of independence and the Mann-Whitney U test were employed to compare participant characteristics and scores between the two groups. Pre- and post-intervention score differences were analyzed using the Wilcoxon signed-rank test. Furthermore, linear mixed models were applied to investigate the interaction effects of the therapeutic intervention on quantitative date score changes. Statistical significance was set at p < 0.05.

## Results

In total, 42 participants (17 male, 25 female) were included in this study, equally divided between the two groups. Sample characteristics can be found in Table 1. Baseline characteristics were similar between the two groups, with no statistically significant differences in age, gender, BMI, occupation type, comorbidities, or baseline outcome measures (Table 2). This suggests that any post-intervention differences can be attributed to the intervention rather than pre-existing differences between groups.

**Table 2:** Baseline measurements of the outcome measures for Group A and Group B RMDQ: Roland Morris Disability Questionnaire, SFMPQ: Short-Form McGill Pain Questionnaire, VAS: Visual Analogue Scale, TSK: Tampa Scale of Kinesiophobia

Baseline Measurements	Group A	Group B	U – Statistic	p-value
Disability (RMDQ)	12.8(6.4)	12.1(5.3)	257.0	0.830
SFMPQ				
VAS	3.62 (0.86)	4.05(0.97)	125.0	0,139
Sensory subscale	13.9(5.8)	14.9(5.8)	123.0	0.551
Affective subscale	9.0(3.1)	10.0(3.6)	117.0	0.361
Total Score	22.9(8.0)	24.9(8.5)	179.0	0.465
TSK Kinesiophobia	22.3(8.7)	19.2(5.8)	285.0	0.279
Proprioception				
Open Eyes	160.9(67.9)	219.3(192.7)	120.0	0.232
Closed Eyes	242.9(121.9)	369.3(506.3)	95.0	0.571

Significant within-group improvements were observed in both intervention groups across all measured outcomes (Table 3). Participants in both the Qigong and Standard Exercises groups demonstrated reductions in disability (RMDQ scores), pain intensity (VAS and SFMPQ), sensory and affective pain subscales, total pain scores, kinesiophobia (TSK), and proprioception with both eyes open and closed (p < 0.05 for all measures).

**Table 3:** Before and after treatment differences of the outcome measures for Group A and Group B RMDQ: Roland Morris Disability Questionnaire, SFMPQ: Short-Form McGill Pain Questionnaire, VAS: Visual Analogue Scale, TSK: Tampa Scale of Kinesiophobia

-	Before	After		
	Mean (SD)	Mean (SD)	W- Statistic	p-value
Group A (Qigong)				
Disability (RMDQ)	12.8(6.4)	4.7(5.7)	18.0	<0.001
SFMPQ VAS	3.62(0.86)	0.95(0.74)	0.0	<0.001
Sensory subscale	13.9(5.8)	5.2(5.0)	22.0	<0.001
Affective subscale	9.0(3.1)	2.4(2.7)	13.0	<0.001
Total Score	22.9(8.0)	7.6(7.2)	14.0	<0.001
TSK	22.3(8.7)	13.0(5.8)	7.0	<0.001
Proprioception				
Open Eyes	160.9(67.9)	123.7(40.5)	75.0	<0.001
Closed Eyes	242.9(121.9)	192.1(84.3)	87.0	0.005
Group B (Standard Exercises)				
Disability (RMDQ)	12.1(5.3)	5.2(4.4)	5.0	<0.001
SFMPQ				
VAS	4.05(0.97)	1.29(0.78)	1.0	<0.001
Sensory subscale	3.62(0.86)	0.95(0.74)	0.0	<0.001
Affective subscale	13.9(5.8)	5.2(5.0)	1.0	<0.001
Total Score	24.9(8.5)	7.7(7.1)	0.0	<0.001
TSK	19.2(5.8)	13.3(7.2)	17.0	<0.001
Proprioception				
Open Eyes	219.3(192.7)	121.0(52.1)	47.0	<0.001
Closed Eyes	369.3(506.3)	200.6(117.8)	73.0	<0.001

However, no significant between-group differences were observed for between-group differences (Table 4). More specifically, both emotional pain and sensory pain scores as well as total pain scores and VAS scale, and proprioception scores with eyes closed and open and disability all always showed similar improvements in both intervention groups (p>0.05). The same was observed for kinesiophobia even though the p-value observed was 0.069, indicating a trend towards significance.

**Table 4:** Between-group differences before and after treatment RMDQ: Roland Morris Disability Questionnaire, MPQ: McGill Pain Questionnaire, VAS: Visual Analogue Scale, TSK: Tampa Scale of Kinesiophobia

Measure	Group A (Pre) Mean (SD)	Group A (Post) Mean (SD)	Group B (Pre) Mean (SD)	Group B (Post) Mean (SD)	F-Value	Interaction p-value
Disability (RMDQ)	12.8 (6.4)	4.7 (3.7)	12.1 (5.3)	5.2 (4.4)	15.6	0.532
MPQ Pain (Total)	22.9 (8.0)	7.6 (7.2)	24.9 (8.5)	7.7 (7.1)	21.94	0.4
VAS	3.62(0.86)	0.95(0.74)	4.05(0.97)	1.29(0.78)	70.82	0.764
Sensory subscale	3.62(0.86)	0.95(0.74)	3.62(0.86)	0.95(0.74)	85.74	0.456
Affective subscale	13.9(5.8)	5.2(5.0)	13.9(5.8)	5.2(5.0)	14.38	0,519
TSK Kinesiophobia	22.3 (8.7)	13.0 (5.8)	19.2 (5.8)	13.3 (7.2)	9.58	0.069
Proprioception						
Open Eyes	160.9(67.9)	123.7(40.5)	219.3(192.7)	121.0(52.1)	7.22	0.136
Closed Eyes	242.9(121.9)	192.1(84.3)	369.3(506.3)	200.6(117.8)	21.76	0.211

## Discussion

The aim of this study was to investigate the effectiveness of a standardized Qigong program versus a standardized strengthening exercise both combined with conventional physiotherapy for patients with chronic nonspecific low back pain. The results of this study indicate that both intervention programs led to significant improvements for the primary (pain) and the secondary outcome measures compared to baseline (pre- and post-treatment/within-groups differences). There were no statistically significant differences observed between the two groups.

Pain perception

Pain perception, assessed by the Short Form McGill Pain Questionnaire, showed significant improvement in both groups before and after treatment. Although the improvement in both groups cannot be attributed separately either for the physiotherapy or the exercise/Qigong regimes, it is interesting to notice that physiotherapy programs like the one it was implemented in this study have a beneficial effect on CLBP conditions [17,18]. On the other hand, the effectiveness of exercise in reducing pain on CLBP patients has been documented by various exercise programs similar to the one it was employed in this study [19-22]. Similarly, participants in the physiotherapy plus Qigong group also experienced a significant decrease in pain perception, supporting findings from studies assessing the effect of Qigong in pain management [23,24]. These findings highlight that both strengthening exercises and Qigong are effective interventions for reducing pain perception when combined with physiotherapy, offering valuable options for pain management in clinical practice. 

Kinesiophobia

Both intervention groups exhibited high kinesiophobia scores at baseline, consistent with existing literature on the psychological impact of chronic low back pain [25]. Following the interventions, both groups demonstrated significant reductions in kinesiophobia scores. Although the Qigong group demonstrated a greater reduction, this difference did not reach statistical significance (p=0.069) and should be interpreted with caution. The observed trend may be related to the meditative and cognitive elements embedded in Qigong practice. According to the literature the additional benefits provided by Qigong in reducing fear of movement and kinesiophobia may enhance overall functional recovery and quality of life [26,27]. Future studies with larger samples and longer follow-up periods are warranted to explore this potential further.

Disability and functionality

Disability levels, as measured by the RMDQ, were of high values at baseline for both groups, reflecting the impact of chronic low back pain on daily activities such as walking, dressing, and work-related tasks [28]. After the interventions, participants in both groups reported improved functionality (within-group difference), but with no difference between groups. These results align with previous studies showing the benefits of exercise programs and physiotherapy for individuals with chronic low back pain [29-31]. The Qigong group demonstrated equal improvements in functionality, suggesting that the combination of Qigong with physiotherapy may offer additional benefits in enhancing physical capabilities and reducing disability, similar to standard exercise.

Proprioception and balance

It was assumed that the group following the therapeutic regime of Qigong exercises due to their balance and proprioception parameters would stimulate more significant results in balance and proprioception compared to the static strengthening group. The existing research suggests that chronic low back pain impairs proprioceptive structures, leading to reduced muscle responses and balance deficits [32,33]. Proprioceptive function, assessed through sway measurements, showed improvements in both groups, without any differences between them. In this study participants exhibited reductions in anteroposterior and lateral sway, indicating improved balance. Studies in the past have demonstrated positive outcomes in proprioception, both when physiotherapy was combined with exercise programs and when standalone strengthening or Qigong exercise programs were employed [34-36].

Clinical implications

These findings suggest that Qigong may be considered as an alternative to traditional strengthening exercises in physiotherapy programs for CNSLBP. Given its potential to reduce kinesiophobia, it may be particularly beneficial for patients exhibiting high movement-related fear. Additionally, Qigong’s low physical demand and adaptability make it a viable option for older adults or individuals with comorbid conditions. Integrating Qigong into rehabilitation settings may require specialized instructor training, which should be considered in future implementation studies.

Strengths and limitations

Among this study’s strengths are the randomized controlled design and the use of validated outcome measures that enhance the validity and reliability of the study. Another strength is the pragmatic design of the physiotherapy and exercise program, which incorporates both supervised and home-based exercises, enhancing its clinical applicability. [22]. In this study, comprehensive assessments of pain, disability, kinesiophobia, and proprioception were also followed, addressing all possible aspects of Qigong as a holistic intervention [8].

Among the limitations of this study is that the short intervention duration (four weeks) may have limited the full therapeutic effects, as exercise-based interventions often require 12 weeks to achieve optimal effects [5,6]. Also, the lack of a physiotherapy-only control group and the absence of a long-term follow-up restrict the generalizability of the study. Additionally, unrecorded patient compliance may have contributed to variability, and the study may have been underpowered to detect between-group differences in kinesiophobia (p=0.069). Future research should include longer interventions, follow-up assessments, and larger sample sizes.

## Conclusions

This study aimed to explore the effect of Qigong on low back pain when combined with standardized physiotherapy, in comparison to conventional strengthening exercises combined with standardized physiotherapy. The results demonstrated that both intervention programs led to significant improvements in pain perception, kinesiophobia, functionality, and proprioception pre- and post-treatment (within-groups differences). However no statistically significant differences could be observed between the two groups. A trend revealed in this study for the Qigong group to improve kinesiophobia further than the strengthening programs requires to be confirmed and replicated with a specific trial designed to investigate the psychological effects of Qigong therapeutic regime in an extended time frame.

## Appendices

Appendix 1: Strengthening exercise Programme

Initial Sessions (Sessions 1-2):

1. Abdominal Exercises (supine position):  

   - Lie on the back with a pillow under the lumbar region.  

   - Perform knee flexion while pressing into the pillow.  

   - 10 repetitions, 3 sets with 20-second rest between sets.  

2. Gluteal Exercises (supine position):  

   - Lift pelvis off the ground, repeating 10 times for 2 sets with the same rest period.  

3. Dorsal (Back) Exercises (prone position):  

   - Lying face down, lift torso (shoulders raised). 

   - Perform 10 repetitions for 2 sets, with rest between sets.  

4. Stretching: 

   - Stretch the targeted muscle groups at the end of each session.

Initial Progression (Sessions 3-4):

1. Abdominal Exercises:  

   - Perform the same supine exercises, but now lift the head and shoulders off the ground.  

2. Gluteal Exercises:  

   - Same pelvic lifting, but now perform it on one leg (alternating legs).  

3. Dorsal Exercises:  

   - In the prone position, lift the right leg with the left arm (and vice versa) and hold for 10 seconds.  

   - Repeat 3 times.

Final Progression (Sessions 5-8):

1. Abdominal Exercises:   

   - Perform supine exercises while pulling an elastic band with the hands and lifting the torso.  

2. Gluteal Exercises:  

   - Same as before  

3. Dorsal Exercises:  

   - Raise both upper and lower limbs in the prone position, holding for 10 seconds, 3 repetitions.  

4. Stretching: 

   - Stretching as in stage 1.

 Structure and Frequency:

- Sessions: 8 total, with 2 supervised sessions per week.  

- Home Practice: Daily exercises at home, with additional sessions twice a week.  

- Total Exercise Time: 20-30 minutes per session, adding up to 40-60 minutes weekly. 
